# Benchmarking Gemini 1.5 Pro in Pediatric Emergency Radiology: Sensitivity Gap and Physiological Bias in the Immature Skeleton

**DOI:** 10.3390/diagnostics16193112

**Published:** 2026-09-24

**Authors:** Cristiano Benelli, Luca Labianca, Vito Caiolo, Gabriele Altavilla, Nicola Maffulli

**Affiliations:** Orthopedic Unit, Sant’Andrea Hospital, University of Rome La Sapienza, Via di Grottarossa 1035, 00189 Rome, Italy; cristiano.benelli@uniroma1.it (C.B.); luca.labianca@uniroma1.it (L.L.); vito.caiolo@uniroma1.it (V.C.); gabriele.altavilla@uniroma1.it (G.A.)

**Keywords:** large language model (LLM), pediatric fractures, artificial intelligence (AI), skeletal maturity, Gemini 1.5 Pro, clinical decision support systems (CDSS)

## Abstract

**Background/Objectives:** To assess the diagnostic performance of Gemini 1.5 Pro, a general-purpose Large Multimodal Model (LMM), as a zero-shot, smartphone-based decision support tool to distinguish pediatric fractures from healthy controls. **Methods:** A retrospective diagnostic accuracy study was conducted on 182 pediatric radiographs (112 confirmed fractures, 70 healthy controls) using the Gemini 1.5 Pro mobile interface with a 4-question prompt protocol (Detection, Characterization, Skeletal Maturity, Management). Diagnostic metrics were calculated with 95% Confidence Intervals (95% CI). Ground truth was established by a senior musculoskeletal radiologist. **Results:** The model showed an overall sensitivity of 90.2% (95% CI: 83.1–95.0%) and specificity of 87.1% (95% CI: 77.0–93.9%). A significant sensitivity gap occurred between displaced (100.0%; 95% CI: 91.4–100.0%) and non-displaced fractures (84.5%; 95% CI: 74.0–92.0%; *p* = 0.007). Qualitative taxonomy revealed a primary physiological bias leading to a 12.8% false-positive rate due to growth plate misinterpretation, alongside minor characterization and localization errors (3.6% of correctly identified fractures). When fractures were correctly identified, proposed management strategies aligned with international standards in 95.0% of cases. Inter-observer reliability was excellent (weighted k = 0.96). **Conclusions:** Gemini 1.5 Pro displays robust clinical reasoning for obvious trauma. However, its zero-shot performance is limited by subtle morphological disruptions and developmental anatomical nuances such as open growth plates. It shows promise as a mobile clinical decision support system for non-specialists, but expert human supervision remains necessary to ensure safety in the complex radiographic landscape of the immature skeleton.

## 1. Introduction

Artificial Intelligence (AI) has advanced from simple computational systems to complex architectures that can emulate human cognitive functions [1]. The field includes machine learning (ML), deep learning (DL), and computer vision (CV) [2,3]. The emergence of Generative AI (GAI) and Large Multimodal Models (LMMs) represents a recent shift in healthcare [4,5]. Unlike traditional convolutional neural networks (CNNs), which are designed for specific tasks, LMMs such as Gemini 1.5 Pro combine natural language processing with advanced visual understanding. This has changed diagnostic and therapeutic workflows and may act as clinical decision support systems (CDSS) [6]. In orthopedic surgery, AI-driven tools are increasingly used to improve clinical outcomes [7]. Digital platforms require new methods to ensure trustworthy and accessible patient education and clinical information [8]. However, integration into routine practice remains complex because of ethical concerns, algorithmic bias, and the need for clinical validation [9]. These models can be used in pediatric trauma care within emergency settings. Pediatric fractures present diagnostic challenges given the plasticity of young bone and the presence of open growth plates (physes). These factors result in injury patterns, such as greenstick or torus fractures, that differ fractures in adults’ cases [10]. AI models have shown promise, but they often need significant computational resources and specific integration. General-purpose LMMs offer a “zero-shot” capability that can be accessed via mobile devices, potentially serving as a “safety net” for clinicians. The ability of LMMs to interpret the radiographic features of the immature skeleton remains unproven [11]. Most literature focuses on task-specific AI models. There is a knowledge gap regarding the “zero-shot” diagnostic performance and specificity of general-purpose multimodal models in a point-of-care, smartphone-based context. The aim of this study was to evaluate the diagnostic accuracy, specificity, and therapeutic reliability of Gemini 1.5 Pro in a cohort of pediatric fractures compared to healthy controls. The hypothesis was that the model might excel in identifying displaced fractures. Its sensitivity would decrease in non-displaced cases. Its specificity would be challenged by the interpretation of skeletal maturity (growth plates) when compared to the gold standard of experienced pediatric orthopedic surgeons.

## 2. Methods

### 2.1. Study Design and Sample Selection

A retrospective diagnostic accuracy study was conducted on radiographic images acquired between June and October 2025 at [Sant’Andrea University Hospital, Sapienza University of Rome, Rome, Italy]. The study followed the STARD 2015 (Standards for Reporting Diagnostic Accuracy) guidelines [12]. To eliminate potential selection bias and provide a robust assessment of model specificity, the dataset was composed of a total of 182 pediatric radiographs obtained from 182 unique patients, ensuring a strict 1:1 patient-to-image ratio. Only one single radiographic view per individual was included in the study to guarantee complete image independence and eliminate any potential within-patient statistical clustering bias. This cohort included 112 fractures (including greenstick, torus, and displaced patterns) and 70 healthy controls (negative for fractures), all confirmed by a senior consultant radiologist with expertise in musculoskeletal imaging. To guarantee a robust reference standard, the radiologist did not evaluate the images in isolation; instead, baseline radiographic features were comprehensively integrated with Emergency Department clinical records and physical examination findings. For ambiguous or subtle non-displaced cases (such as torus or greenstick injuries), the final ground truth was verified by reviewing subsequent clinical and radiographic follow-up data obtained 10–14 days post-injury to confirm the presence of healing (e.g., periosteal reaction or callus formation) or the uneventful resolution of symptoms. Inclusion criteria focused on upper and lower extremity injuries in patients under 18 years of age. Cases with doubtful radiographic interpretation or poor image quality were excluded. Fracture displacement was classified according to the AO Pediatric Comprehensive Classification of Long-Bone Fractures (AO/PCCF) [13], with “non-displaced” defined as translation < 2 mm or maintained alignment.

### 2.2. Ethical Considerations and Data Privacy

The study was conducted in accordance with the Declaration of Helsinki. Since this was a retrospective analysis of fully de-identified radiographic images, it was exempt from formal Institutional Review Board (IRB) approval according to local institutional guidelines. Informed consent was obtained from all subjects (or their legal guardians) at the time of original imaging for the use of anonymized data for clinical research purposes. To ensure General Data Protection Regulation (GDPR) compliance and patient privacy, all images were captured from the Picture Archiving and Communication System (PACS) monitor excluding any patient-identifiable metadata (pixel-level de-identification).

### 2.3. AI Evaluation Protocol (Smartphone-Based Point-of-Care)

The Gemini 1.5 Pro mobile interface, using a standard application, simulated a real-world emergency workflow. This workflow required rapid decision support at the bedside. To ensure technical reproducibility, all source radiographs were displayed on a calibrated 5-megapixel medical diagnostic monitor (Barco MDCC-6530; Barco NV, Kortrijk, Belgium) through the institutional PACS software (Vue PACS version 12.2; Carestream Health, Inc., Rochester, NY, USA). Images were captured using contemporary commercial smartphones (iPhone 14; Apple Inc., Cupertino, CA, USA, and Samsung Galaxy S23; Samsung Electronics Co., Ltd., Suwon, South Korea) utilizing their native camera applications at a standardized resolution of 12 megapixels. To minimize optical distortion and external artifacts, all photographs were taken perpendicularly from a fixed distance of approximately 35–40 cm from the screen. The smartphone flash was deactivated, and all acquisitions were performed in dedicated diagnostic rooms under standardized dim ambient lighting conditions to prevent screen glare and moiré patterns. Each radiograph was processed using a Zero-Shot prompting strategy. This strategy assessed the model’s “out-of-the-box” diagnostic and therapeutic reasoning. To replicate the clinical reality of a busy emergency department, each image was queried only once. Iterative prompting does not reflect the time-constrained nature of urgent care.

A standardized 4-question prompt protocol was applied to every case. The protocol was applied verbatim:

1. Detection: “Is there any evidence of a cortical disruption or a fracture line?”
2. Characterization: “How would you describe the fracture morphology and its precise anatomical location?”
3. Skeletal Maturity: “Is the bone skeletally mature or immature? Identify the presence of open growth plates (physes).”
4. Management: “Based on AO/PCCF and NICE guidelines, what is the proposed management strategy: conservative treatment or surgical intervention?”

### 2.4. Scoring and Qualitative Error Assessment

AI responses were quantified using a 3-point Likert scale (0–2) by two blinded investigators, with any scoring discrepancies resolved by a third senior reviewer. Scores were assigned as follows: 0 points for an incorrect response, 1 point for a partially correct or incomplete response, and 2 points for a correct and complete response. The accuracy of the AI-proposed management was benchmarked against established international standards, including the AO/PCCF [13] for morphology, alongside the American Academy of Orthopaedic Surgeons (AAOS) [14] and the National Institute for Health and Care Excellence (NICE) [15] guidelines for therapeutic decision-making.

To provide mechanistic insight into AI failures, a Qualitative Error Taxonomy was implemented:

• Type I (Missed Detection): Failure to identify a cortical disruption (False Negative).
• Type II (Physiological Bias): Misinterpretation of a growth plate or accessory ossicle as a fracture (False Positive).
• Type III (Management Mismatch): Incorrect therapeutic suggestion despite correct fracture identification.
• Type IV (Characterization/Localization Error): Failure to correctly describe fracture morphology or precise anatomical location despite correct fracture identification.

### 2.5. Statistical Analysis

Statistical analysis was performed using IBM SPSS Statistics for Windows, version 28.0 (IBM Corp., Armonk, NY, USA). Diagnostic performance parameters for fracture detection (Q1), including Sensitivity, Specificity, Positive Predictive Value (PPV), and Negative Predictive Value (NPV), were quantified and reported alongside their corresponding 95% Confidence Intervals (95% CI). For ordinal data, namely the Likert scores, results were analyzed descriptively and expressed as Mean ± Standard Deviation (SD). Inter-observer reliability between the blinded investigators was evaluated using the weighted Cohen’s kappa (k) coefficient to account for the ordinal nature of the scoring system. Differences in diagnostic sensitivity between the displaced and non-displaced fracture subgroups were statistically compared using Fisher’s exact test. For all statistical analyses, a *p*-value less than 0.05 was considered to indicate statistical significance.

## 3. Results

### 3.1. Cohort Characteristics and Fracture Distribution

The final dataset comprised 182 pediatric radiographs. The fracture cohort (*n* = 112) had a mean age of 13.05 ± 2.82 years, with a male predominance (73.2%; *n* = 82). The control group consisted of radiographs of 70 healthy pediatric subjects (mean age 12.8 ± 3.1 years) in whom no evidence of a fracture was present. The anatomic distribution and fracture characteristics (displaced vs. non-displaced) are detailed in Table 1.

### 3.2. Global Diagnostic Performance and Metrics

The diagnostic performance of the model for fracture detection (Q1) is summarized in Table 2. The model demonstrated an overall sensitivity of 90.2% (95% CI: 83.1–95.0%) and a specificity of 87.1% (95% CI: 77.0–93.9%). The Positive Predictive Value (PPV) was 91.8% (95% CI: 85.0–95.7%) and the Negative Predictive Value (NPV) was 84.7% (95% CI: 74.3–91.5%). Inter-observer reliability for the scoring process yielded a weighted Cohen’s kappa (k) of 0.96, indicating almost perfect agreement.

### 3.3. Task-Specific Likert Analysis

Model performance was task-dependent, with mean Likert scores (0–2) reflecting varying levels of reliability across the four clinical queries (Table 3):

Q1 (Detection): 1.80 ± 0.60
Q2 (Morphology): 1.73 ± 0.65
Q3 (Skeletal Maturity): 1.52 ± 0.83
Q4 (Management): 1.71 ± 0.66

### 3.4. Subgroup Sensitivity Analysis

Fracture displacement significantly influenced diagnostic sensitivity (Fisher’s Exact Test, *p* = 0.007). The model achieved ceiling performance with a sensitivity of 100% (41/41; 95% CI: 91.4–100.0%) for displaced fractures. Sensitivity significantly dropped to 84.5% (60/71; 95% CI: 74.0–92.0%) in non-displaced cases (Fisher’s Exact Test, *p* = 0.007). No adjustments for multiple comparisons were applied, as this subgroup stratification was pre-specified in the primary study protocol based on our clinical hypothesis. Most missed detections (Type I errors) were associated with subtle greenstick or minimally displaced lesions.

### 3.5. Qualitative Error Taxonomy

Analysis of model failures across the entire dataset (*n* = 16 errors in the fracture group; *n* = 9 errors in the control group) revealed the following distribution:

Type I (Missed Detection): 11 cases (6.0% of the total cohort). These were predominantly non-displaced fractures of the wrist and distal tibia within the fracture group (Figure 1).

Type II (Physiological Bias): 9 cases (12.8% of the control group). All cases involved the model erroneously identifying an open physis (growth plate) as a cortical disruption in healthy patients (Figure 2).

Type III (Management Mismatch): 5 cases (5.0% of correctly identified fractures; 5/101). These involved suggestions for conservative treatment in cases where AO/PCCF and NICE guidelines indicated surgical stabilization due to angulation limits.

Type IV (Characterization/Localization Error): 4 cases (3.6% of correctly identified fractures). In these instances, the model successfully detected the cortical disruption (Q1) but failed to pinpoint the exact bone involved (e.g., mistaking the radius for the ulna) or mischaracterized the structural pattern (e.g., confusing a greenstick fracture with a complete transverse fracture).

**Figure 1:**
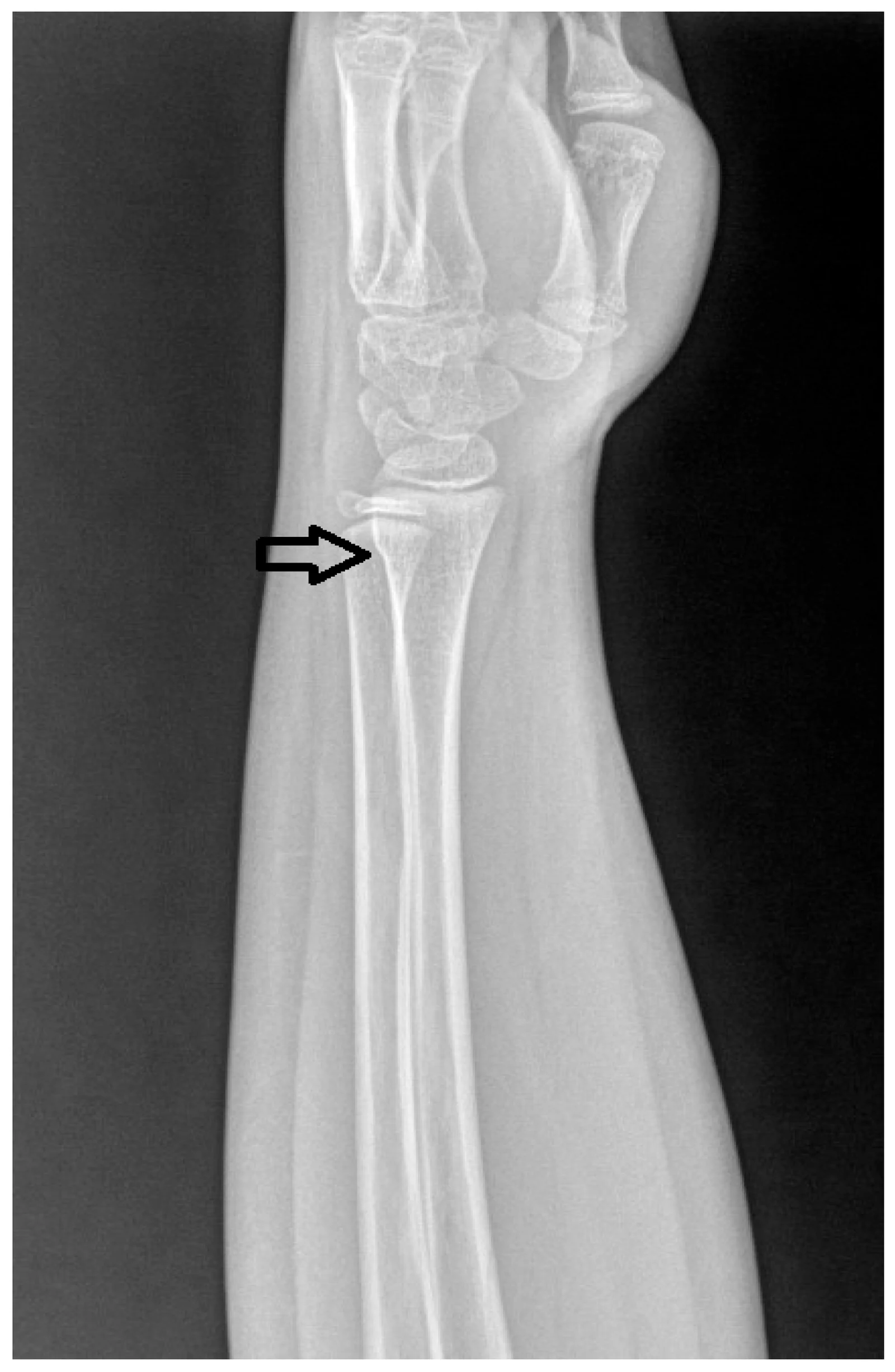
Example of a Type I Error (Missed Detection/False Negative) in a pediatric wrist radiograph. The blackarrow indicates a subtle, non-displaced torus (buckle) fracture of the distal radius cortex. Reference Diagnosis: Acute non-displaced distal radius torus fracture. Model Verbatim Interpretation: “Negative for cortical disruption. Cortical margins appear intact; no definitive evidence of an acute fracture line or displacement visualized”.

**Figure 2:**
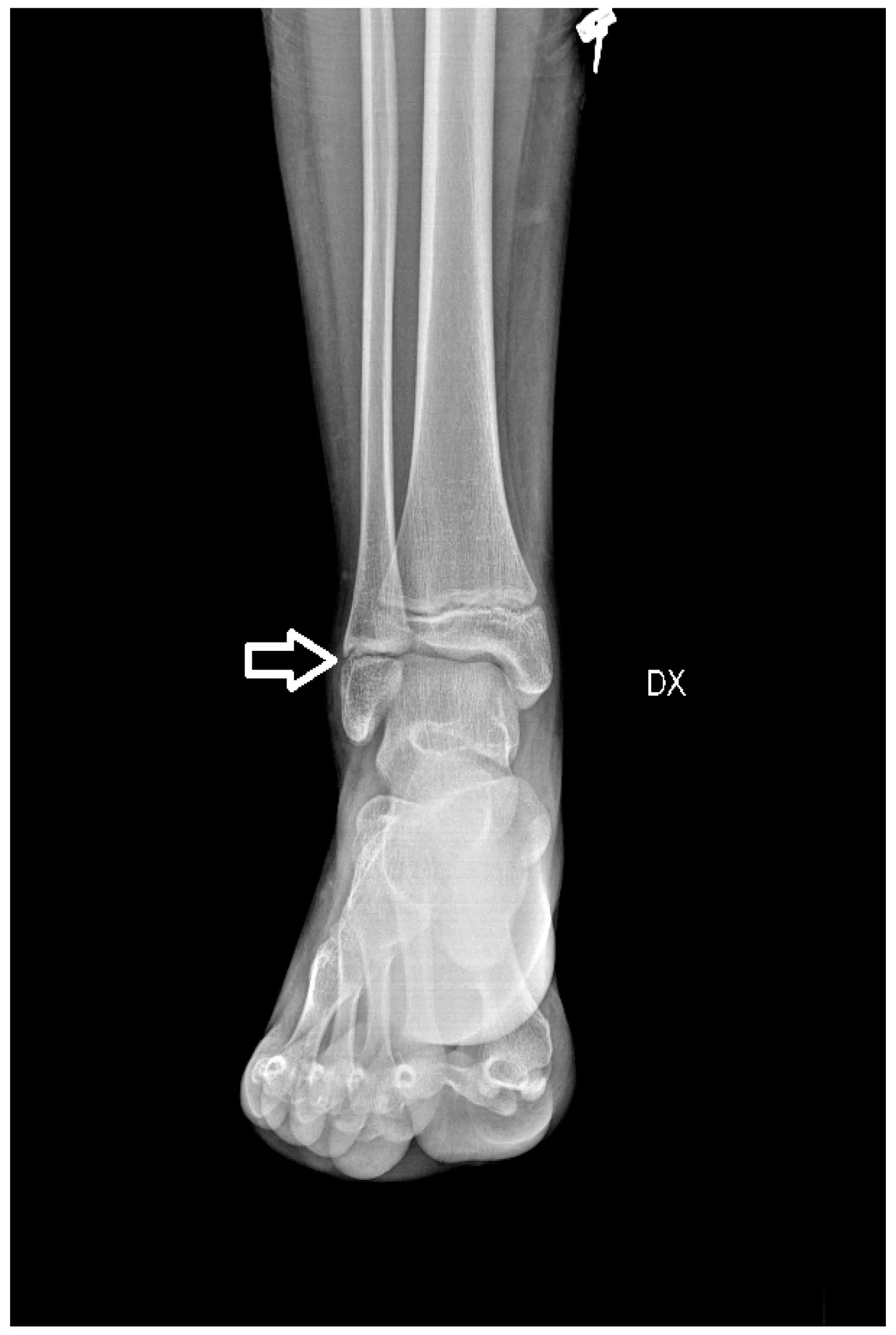
Example of a Type II Error (Physiological Bias/False Positive) in a healthy pediatric ankle radiograph. The whitearrow highlights a normal, open distal fibula growth plate (physis). Reference Diagnosis: Healthy control ankle, negative for acute fractures. Model Verbatim Interpretation: “Positive for cortical disruption. Evidence of a non-displaced fracture line involving the lateral malleolus”.

## 4. Discussion

Gemini 1.5 Pro achieved strong diagnostic performance for pediatric fractures, with 90.2% sensitivity and 87.1% specificity. Including a balanced control group (*n* = 70) offered a more realistic assessment of the model’s usefulness in a clinical setting, consistent with calls for rigorous external validation when benchmarking such models. The model performed exceptionally well in identifying displaced fractures (100%). However, it showed a significant “sensitivity gap” in non-displaced patterns (84.5%; *p* = 0.007).

This diagnostic pattern highlights the operational nuances of general-purpose LLMs when compared to task-specific architectures, as recently demonstrated by Haupt et al. (2026) [16]. In their evaluation of zero-shot multimodal models on the GRAZPEDWRI-DX dataset for pediatric wrist fractures, they reported a lower patient-level accuracy of approximately 0.55, which is only marginally better than chance. This apparent discrepancy with our higher overall metrics can be directly attributed to structural differences in dataset composition, image characteristics, and task methodology. First, while the dataset used by Haupt et al. consisted primarily of subtle, non-displaced distal radius and ulna lesions (buckle/torus fractures accounted for 79% of their cohort), our real-world clinical sample included a substantial proportion of displaced fractures (36.6%), where Gemini achieved a ceiling sensitivity of 100%. When isolating our non-displaced subgroup, our sensitivity drops to 84.5%, echoing the model vulnerabilities in subtle patterns identified in their literature. Second, the method of image input plays a pivotal role; direct API feeding of high-resolution native files often forces models to aggressively downsample images, losing fine cortical details. Conversely, our smartphone-captured point-of-care approach introduced an implicit human-in-the-loop attention mechanism, where the operator pre-cropped and centered the relevant anatomical region on the PACS monitor, effectively reducing visual noise. Finally, while Haupt et al. tested models on strict spatial localization tasks (bounding boxes), our primary detection metric relied on a binary textual query, better aligning with the internal probabilistic knowledge processing of foundational architectures.

The discrepancy of our findings underscores the fundamental architectural divergence between general-purpose LLMs and task-specific, supervised Convolutional Neural Networks (CNNs) in musculoskeletal imaging. While foundational LLMs process visual data by converting images into flattened patches or visual tokens embedded within a massive cross-modal text-image knowledge base, traditional CNNs operate via localized pixel-level spatial convolutions optimized purely for geometric and contrast-based feature extraction. Consequently, specialized CNN architectures (such as YOLOv8 or customized ResNet models) [17,18] demonstrate far greater pixel-level sensitivity and spatial precision when identifying minute, high-frequency structural disruptions, such as subtle pediatric cortical buckle or non-displaced greenstick fractures. Furthermore, CNNs exhibit absolute determinism, ensuring that identical diagnostic pixels strictly yield identical classification outputs, which is critical for regulatory compliance and safety-critical medical devices. However, the structural limitation of task-specific CNNs lies in their complete absence of clinical contextualization; they function as isolated image classifiers that output binary probabilities or localized bounding boxes without any capacity for clinical reasoning, multi-class integration, or evidence-based guideline alignment. Conversely, general-purpose LLMs, despite their current visual tokenization ceilings and susceptibility to physiological hallucinations such as growth plate misinterpretations [9,19], exhibit an internalized clinical knowledge base capable of acting as an advanced Clinical Decision Support System (CDSS). As observed in the present study, once a fracture is detected, LLMs can natively bridge visual inputs with sophisticated evidence-based therapeutic recommendations (e.g., AO/PCCF and NICE guidelines) [13,15], a task that standard computer vision networks cannot perform without extensive, multi-modal pipeline engineering.

Gemini 1.5 Pro exhibited a consistent error pattern: mistaking open growth plates for fractures. On the skeletal-maturity question (Q3), it scored lowest and varied most. This “physiological bias” produced a 12.8% false-positive rate in healthy children: the model essentially saw a normal physis and classified it as a fracture. This is a real limitation for any tool meant to support emergency decisions. This aligns with recent benchmarks evaluating alternative LMMs in musculoskeletal imaging [19,20]. For instance, a recent investigation [20] of a multimodal model (ChatGPT-4o) in the ossifying pediatric elbow reported similar diagnostic hurdles, specifically regarding the differentiation between normal developmental anatomical variations and subtle, non-displaced fractures. Comparative analyses [19] of multimodal foundational architectures on diagnostic radiology examinations have highlighted that both Gemini and GPT frameworks experience similar performance ceilings when interpreting complex radiological structures without domain-specific fine-tuning. This consistent limitation across different foundational models confirms that the unique complexity of pediatric imaging is due to the inherent plasticity and open physes of the immature skeleton, which remains a primary challenge for general-purpose computer vision [18,21]. Consequently, this differentiation remains the “gold standard” challenge in pediatric orthopedics.

Compared to dedicated musculoskeletal systems [22], multi-class deep learning models calibrated for specific anatomic regions such as the pediatric distal forearm [17], or radiomics-based models [23] that achieve high accuracy in specific anatomic districts, Gemini’s performance shows that general-purpose computer vision is not yet fully calibrated to the developmental stages of the human skeleton. However, the results show a notable strength in clinical management (Q4). Here, the model showed high alignment with AO/PCCF and NICE guidelines. This suggests that the model’s internalized knowledge base successfully integrates evidence-based standards, positioning it as a sophisticated CDSS rather than a simple image classifier.

The introduction of a Type IV error classification highlights that, while generative models possess a high semantic baseline for trauma detection, fine-grained anatomical and morphological characterization remains a challenge. Misidentifying adjacent long bones or misclassifying pediatric-specific fracture patterns (such as torus or greenstick variations) underscores the necessity of domain-specific calibration before these systems can be safely utilized as autonomous diagnostic aids.

A unique aspect of this study is the evaluation of the smartphone-based “point-of-care” workflow. Most AI research in radiology assumes direct integration into the PACS [9,23]. However, the “Zero-Shot” mobile approach reflects the reality of frontline clinicians who may use portable devices for quick consultation. While this decentralized use-case offers unprecedented accessibility, it introduces challenges related to image resolution and data privacy. Even with images taken on smartphones, the model maintains a high Negative Predictive Value (84.7%), indicating its potential as a “safety net” for non-expert clinicians in emergency settings.

Within a medical system infrastructure, the incorporation of a balanced cohort is fundamental to accurately evaluating the false-positive rate [24]. A lower specificity, such as the 87.1% observed in the cohort, directly translates into clinical alert fatigue for emergency physicians, who must constantly filter out erroneous machine suggestions [25]. Benchmarking indicates that, while Gemini 1.5 Pro provides a “logic-driven” orthopedic consultation, its specificity is currently limited by the lack of age-specific anatomical “priors.” Future “Medical Systems” integration should consider including patient data like age and clinical history to reduce Type II errors. Ultimately, Gemini 1.5 Pro may serve as a robust CDSS, provided that a human specialist remains the final “filter” to interpret the subtle features of the developing skeleton. This observation is consistent with the view that AI assistance can effectively elevate the overall diagnostic performance of clinical residents [10].

### Limitations

This study has several limitations. For example, while the inclusion of 70 healthy controls addressed previous concerns regarding selection bias, the total sample size (*n* = 182) remains a pilot cohort for general-purpose model validation. However, this sample was methodologically sufficient to achieve statistical significance (*p* = 0.007) regarding sensitivity differences and to identify specific “Physiological Bias” patterns in pediatric anatomy. Also, the “Zero-Shot” approach, while simulating a real-world “point-of-care” scenario, does not explore more advanced prompting techniques such as “Few-Shot” learning or “Chain-of-Thought” reasoning, which could potentially improve diagnostic specificity. Furthermore, we assessed the model’s performance using a single-query session per image. While this single-query design introduces limitations regarding statistical reproducibility from the inherent stochasticity of generative outputs, it was a deliberate methodological choice to replicate the time-constrained reality of urgent care. Frontline emergency clinicians require rapid, single-session point-of-care decision support and lack the operational time for iterative prompt engineering or multi-session verification. To control model drift during the study period, all evaluations were conducted using the exact same stable production release of the Gemini 1.5 Pro interface (2024 architecture). Nonetheless, this approach stands as a limitation, and future studies should perform multiple iterations to quantify the inter-session reliability of the model. The reliance on smartphone-captured images from a PACS monitor, although reflecting a common “ubiquitous computing” practice in hospitals, introduces variables such as screen glare and resolution loss. This “pixel-level” limitation may have contributed to the Type I errors (missed detections) observed in non-displaced fractures. The internal reasoning processes of LLMs are not fully understood because they are “black box” systems. Although a Qualitative Error Taxonomy provided mechanistic insight, model performance depends on the specific Gemini 1.5 Pro architecture version and may well change with updates. Finally, the retrospective, single-center design does not reflect the dynamic, multi-modal nature of emergency care. In this setting, surgeons use imaging with physical examination and longitudinal clinical context. Future studies should investigate integrating patient metadata, such as age and clinical history, into the prompt. This could assess if such context can mitigate the “Growth Plate Bias” identified in this study.

## 5. Conclusions

This study suggests that a LLM has the potential to assist in identifying pediatric fractures, providing valuable morphological characterizations and evidence-based management suggestions in many instances. However, notable domain-specific vulnerabilities appear to persist, particularly regarding the sensitivity gap observed in non-displaced fractures and a potential physiological bias when assessing skeletal maturity. Therefore, while general-purpose LLMs may serve as supplementary screening tools, they should not be used autonomously, especially in pediatric imaging, where expert oversight remains essential, and is particularly evident in the complex radiographic landscape of the developing skeleton. Future research could beneficially focus on fine-tuning these models with broader, domain-specific datasets, or exploring hybrid architectures that combine the orthopedic logic of LLMs with the precise pattern recognition of specialized convolutional neural networks to better evaluate their safety and effectiveness in clinical integration.

## Figures and Tables

**Table 1:** Clinical and Demographic Characteristics of the Pediatric Study Population.

Characteristic	Fracture Cohort (*n* = 112)	Healthy Controls (*n* = 70)	Total Dataset (*n* = 182)
**Demographic Profiles**			
Age (years), Mean ± SD	13.05 ± 2.82	12.80 ± 3.10	12.95 ± 2.93
Gender, *n* (%)			
Male	82 (73.2%)	48 (68.6%)	130 (71.4%)
Female	30 (26.8%)	22 (31.4%)	52 (28.6%)
**Anatomic Site (Fracture Cohort Only)**, *n* (%)			
Wrist	30 (26.8%)	N/A	30 (16.5%)
Tibia (plateau, shaft, malleolus)	26 (23.2%)	N/A	26 (14.3%)
Hand (phalanges, metacarpals)	21 (18.8%)	N/A	21 (11.5%)
Clavicle	15 (13.4%)	N/A	15 (8.2%)
Elbow (distal humerus, radial head)	11 (9.8%)	N/A	11 (6.0%)
Foot (phalanges, metatarsals)	4 (3.6%)	N/A	4 (2.2%)
Ulnar shaft	4 (3.6%)	N/A	4 (2.2%)
Femur	1 (0.8%)	N/A	1 (0.5%)
**Fracture Displacement (Fracture Cohort Only)**, *n* (%)			
Non-displaced	71 (63.4%)	N/A	71 (39.0%)
Displaced	41 (36.6%)	N/A	41 (22.5%)

Note: SD = Standard Deviation; N/A = Not Applicable. Percentages may not total 100% due to rounding.

**Table 2:** Diagnostic Performance Metrics of Gemini 1.5 Pro for Pediatric Fracture Detection.

Metric	Value	95% Confidence Interval (95% CI)
**Sensitivity (Overall)**	90.2% (101/112)	83.1–95.0%
*Displaced Subgroup*	100.0% (41/41)	91.4–100.0%
*Non-displaced Subgroup*	84.5% (60/71)	74.0–92.0%
**Specificity**	87.1% (61/70)	77.0–93.9%
**Positive Predictive Value (PPV)**	91.8% (101/110)	85.0–95.7%
**Negative Predictive Value (NPV)**	84.7% (61/72)	74.3–91.5%
**Inter-rater Reliability (Weighted k)**	0.96	0.91–1.00

Note: Sensitivity and Specificity were calculated based on the binary detection of cortical disruption (Question 1) across the total dataset (*n* = 182). Subgroup sensitivities were pre-specified based on fracture displacement criteria. Inter-rater reliability represents the agreement between the two blinded investigators across the ordinal scoring system. Weighted Cohen’s kappa (k) values between 0.81 and 1.00 indicate almost perfect agreement.

**Table 3:** Performance Analysis Stratified by Clinical Task (*n* = 182).

Task ID	Clinical Query	Total Score (Max 364)	Score Attainment Rate (%) *	Mean Score (±SD)
**Q1**	Cortical disruption detection	328	90.1%	1.80 ± 0.60
**Q2**	Morphological description	315	86.5%	1.73 ± 0.65
**Q3**	Skeletal maturity assessment	277	76.1%	1.52 ± 0.83
**Q4**	Management strategy proposal	311	85.4%	1.71 ± 0.66

* Note: Performance was evaluated on a 3-point Likert scale (0: incorrect; 1: partially correct; 2: correct and complete). The Score Attainment Rate is defined as the total points scored divided by the maximum attainable score per task across the entire dataset (*n* = 182). SD = Standard Deviation.

## Data Availability

The data presented in this study are available upon reasonable request from the corresponding author. The radiographic images are not publicly available due to institutional data protection policies, patient privacy regulations, and European GDPR restrictions regarding retrospective clinical diagnostic data.
